# Measurement Uncertainty and Risk of False Compliance Assessment Applied to Carbon Isotopic Analyses in Natural Gas Exploratory Evaluation

**DOI:** 10.3390/molecules29133065

**Published:** 2024-06-27

**Authors:** Fabiano Galdino Leal, Alexandre de Andrade Ferreira, Gabriel Moraes Silva, Tulio Alves Freire, Marcelo Ribeiro Costa, Erica Tavares de Morais, Jarbas Vicente Poley Guzzo, Elcio Cruz de Oliveira

**Affiliations:** 1 Research Center, Petrobras S.A., Rio de Janeiro 21941-915, Brazil; fabianoleal@petrobras.com.br (F.G.L.); alexandreaf@petrobras.com.br (A.d.A.F.); tulioalves@petrobras.com.br (T.A.F.); marcelo_costa@petrobras.com.br (M.R.C.); ericat@petrobras.com.br (E.T.d.M.); guzzo@petrobras.com.br (J.V.P.G.); 2 Center for Energy Resources Engineering, Department of Chemical and Biochemical Engineering, Technical University of Denmark, 2800 Kongens Lyngby, Denmark; gabsil@kt.dtu.dk; 3 Postgraduate Programme in Metrology, Pontifical Catholic University of Rio de Janeiro, Rio de Janeiro 22451-900, Brazil; 4 Land Transportation and Storage, Measurement and Product Inventory Management, Logistics, Petrobras S.A., Rio de Janeiro 20231-030, Brazil

**Keywords:** biogenic isotopic composition, gas geochemistry, natural gas exploratory evaluation, measurement uncertainty, stable isotope analysis

## Abstract

The concept of uncertainty in an isotopic analysis is not uniform in the scientific community worldwide and can compromise the risk of false compliance assessment applied to carbon isotopic analyses in natural gas exploratory evaluation. In this work, we demonstrated a way to calculate one of the main sources of this uncertainty, which is underestimated in most studies focusing on gas analysis: the δ^13^C calculation itself is primarily based on the raw analytical data. The carbon isotopic composition of methane, ethane, propane, and CO_2_ was measured. After a detailed mathematical treatment, the corresponding expanded uncertainties for each analyte were calculated. Next, for the systematic isotopic characterization of the two gas standards, we calculated the standard uncertainty, intermediary precision, combined standard uncertainty, and finally, the expanded uncertainty for methane, ethane, propane, and CO_2_. We have found an expanded uncertainty value of 1.8‰ for all compounds, except for propane, where a value of 1.6‰ was obtained. The expanded uncertainty values calculated with the approach shown in this study reveal that the error arising from the application of delta calculation algorithms cannot be neglected, and the obtained values are higher than 0.5‰, usually considered as the accepted uncertainty associated with the GC-IRMS analyses. Finally, based on the use of uncertainty information to evaluate the risk of false compliance, the lower and upper acceptance limits for the carbon isotopic analysis of methane in natural gas are calculated, considering the exploratory limits between −55‰ and −50‰: (i) for the underestimated current uncertainty of 0.5‰, the lower and upper acceptance limits, respectively, are −54.6‰ and −50.4‰; and (ii) for the proposed realistic uncertainty of 1.8‰, the lower and upper acceptance limits would be more restrictive; i.e., −53.5‰ and −51.5‰, respectively.

## 1. Introduction

The establishment of quality control protocols for a given analytical technique is crucial to guarantee the reliability of any provided information. This control can be translated into any systematic action that contributes to the increase of confidence in the analytical results and should be consistently associated with cost optimization as part of a robust laboratory management program [1]. Regarding the need to increase the reliability of analytical results, uncertainty measurement is one of the main metrological tools commonly associated with this requirement. Its application in Brazil is based on the international standard for the constant monitoring of data quality by national laboratories [2].

However, when it comes to the isotopic characterization of materials, the concept of uncertainty can be misleading, which can compromise assessments in forensic applications [3]. Most of the uncertainties presented/measured/inferred in previous publications are, in fact, only analytical uncertainties, strictly comprising sample preparation and analysis. For example, in work by Bulska et al. (2015) [4] and Bréas et al. (2007) [5], the authors calculate the expanded uncertainty (“quantity defining an interval about the result of a measurement that may be expected to encompass a large fraction of the distribution of values that could reasonably be attributed to the measurand” [6]) of the technique, but only analytical factors are included, without considering the uncertainty arising from the calculation procedure. This trend is widespread in the stable isotope literature, being reproduced even by manufacturers when reporting “uncertainties” from standards, which are actually experimental deviations from successive repetitions. When conducting a series of repeated analyses, only the variation of the steps between the sample and the integration is obtained. The uncertainty arising from the calculation steps of the $\delta^{13}C\left(‰\right)$ values, which express the abundance of the isotope variation of the ^13^C in a sample, in parts per thousand, and the correction of these values, is poorly discussed. Only a few studies have been dedicated to the evaluation of the expanded uncertainty, considering that the calculation is a source of error as well. Most of the studies transmit the idea that the uncertainty of the analysis is limited to the technique itself, which is imprecise. They do not consider the corrections applied a posteriori to the measurement. This is because these corrections are not effectively introduced in the quality control protocols of the laboratories, as they do not need and/or cannot control the variation of the uncertainty from this source. A few examples of reported uncertainty values from the literature, in several matrices, are shown in Table 1. The range of these uncertainties is extensive, although the different analyzed matrices should be taken into consideration.

This variation can also be related to the calculation method, which will be further discussed. Regarding the isotopic characterization of gases (particularly light hydrocarbons-C_1_ to C_3_-and CO_2_, present in natural gases), there is a broad application related to petroleum exploration and production activities: the origin of these gases (biogenic versus thermogenic), correlate with possible source rocks, the degree of relative thermal evolution, and the possible secondary changes caused by biodegradation, secondary migration, secondary cracking, mixtures, or partial escape from accumulations [31]. This scenario can be more difficult to map when the measured value is overlapped between and close to the lower and upper acceptance limits and/or the measurement uncertainty is large when compared to these acceptance limits. Based on these assumptions, there may be an increased risk of false compliance assessment [32,33]. In other words, when a measurement result accompanied by its expanded uncertainty partially overlap the acceptance limits, the risk of false decisions is increased.

For diagnoses to be valid and support the geochemical interpretations about the gases associated with evaluating a particular petroleum system, it is crucial that the isotopic results are as reliable as possible and have an associated uncertainty compatible with those interpretations. Thus, from this uncertainty information, using the guard bands concept, lower and upper acceptance limits can be proposed to guarantee that there is no risk of false compliance for the carbon isotopic analysis in natural gas—the aim of this study.

## 2. Materials and Methods

In the present study, we have calculated the measurement uncertainty associated with the carbon isotope ratios determination of light hydrocarbons (C_1_–C_3_) and CO_2_ in natural gas samples. We first describe, step by step, all the calculations involved in the uncertainty determination, starting with the ratio between the peak areas of each compound present in the analyzed mixtures until the carbon isotopic ratio of these compounds. We also show the approach used to calculate the uncertainty and describe the analytical method used to analyze the gas samples.

### 2.1. Laboratory Method (GC-IRMS)

Isotope ratio mass spectrometry (IRMS) is used for the measurements of relative abundances of isotopes of an element in a particular sample in such a way that it makes possible the detection of slight differences in those abundances [34,35].

In this study, a gas chromatograph was coupled to the mass spectrometer to allow the previous separation of the components of the gas samples before the conversion of each one to CO_2_ molecules (combustion process), which, in turn, are directed to the isotopic measurements on the mass spectrometer through a continuous flow of helium. In the following section, a brief description of this analytical system is presented.

### 2.2. Test Method

For the carbon isotope analysis of light hydrocarbons in the range of C_1_ to C_3_ and CO_2_, a gas chromatograph (HP 7890 model, Agilent Technologies, Santa Clara, CA, USA) was used. It was equipped with a split/splitless injector (10:1 split ratio, helium flow of 1.7 mL min^−1^) and a Poraplot-Q chromatographic column (10 m × 0.32 mm × 10 μm), coupled to a mass spectrometer for isotopic ratio, model DeltaV Plus (Thermo Fisher Scientific, Waltham, MA, USA), via an interface for combustion maintained at 960 °C. The injections were made manually using syringes and with sample volumes varying between 10 and 200 microliters, according to the relative concentrations of the analytes. The δ^13^C values were calculated concerning the international standard Vienna PeeDee Belemnite (VPDB), using a secondary standard (a natural gas sample), calibrated against a standard prepared by the USGS (United States Geological Survey, Reston, VA, USA), considered a “primary” standard in this study. CO_2_ was used as the reference gas. All analyses were performed in triplicate.

### 2.3. Mathematical Models

In this section, the algorithms involved [35] in the GC-IRMS of the carbon isotope ratios determination in natural gas samples were separated by steps.

**Step 1**. Calculate the isotopic ratios observed among the peak areas, measured by the mass spectrometer. For this stage, one should divide the areas of the following masses (without amplification): 45 by 44 and 46 by 44, respectively, RCO245 and R46CO2; and their respective amplifications, R45CO2observed and R46CO2observed. These values are the variables measured directly by the mass spectrometer via integrating the electronic signal obtained. Alternatively, one can also get the electronic signal measured as a function of time and use its preferred integration method to calculate the areas. The amplifications are calculated according to Equations (1) and (2), considering the amplification constants 100 and 1000/3:

(1) R45CO2observed=R45CO2×100

(2) R46CO2observed=R46CO2×10003

**Step 2**. Provide the $\delta^{13}C$ value of the reference peak. In this case, if the primary standard is being calculated, this value must be adjusted until the value of $\delta^{13}C$ of the standard peak (obtained in step 14), given by the manufacturer, is the desired one. In the case of a sample, the $\delta^{13}C$ value of the reference peak can be previously determined based on a study with a primary standard, and one should just provide it to proceed with the calculations.

**Step 3**. Calculate R13CO2, the carbon isotopic ratio ^13^C to ^12^C for the reference peak, from the equation presented in the methodology for calculating the isotopic ratio, Equation (3):

(3) R13CO2=δ13C1000+1×R13CO2/VPDB

where

▪ R13CO2 is the carbon isotopic ratio in CO_2_;
▪ R13CO2/VPDB is the isotopic ratio of the Vienna Pee Dee Belemnite (VPDB) standard [36].

**Step 4**. Calculate the fractional abundances for carbon 13 and 12, respectively,$13_{F}$ and $12_{F}$, Equations (4) and (5), for the reference peaks:

(4) F13CO2=R13CO2R13CO2+1

(5) F12CO2=1−F13CO2

**Step 5**. Calculate the oxygen isotopic ratios of masses 17 and 18, R17CO2 and R18CO2, respectively, Equations (6) and (7), for the reference peak. Again, using the same mass balances, but this time considering the values of the constants *a* and *K* and the reasons already obtained, one performs the following calculations:

(6) R17CO2=R45CO2−R13CO22

(7) R18CO2=R17CO2K1/a

where

▪ *a* is the regression coefficient of the ratio between the isotopic oxygen ratios 17 and 18 of several international isotopic water standards [34];
▪ *K* is a constant characteristic of the relationship between $17_{R}$ and $18_{R}$ in a terrestrial oxygen reservoir [34].

**Step 6**. Calculate the fractional abundances of oxygens 18, 17, and 16, respectively, F18CO2, F17CO2 and F16CO2, Equations (8)–(10), for the reference peak:

(8) F18CO2=R18CO2R18CO2+1

(9) F17CO2=R17CO2R17CO2+1

(10) F16CO2=1−F18CO2−F17CO2

**Step 7**. Calculate the fractional abundances of isotopes of CO_2_, 44 to 46, respectively, F44CO2, F45CO2 and F46CO2, Equations (11)–(13), for the reference peak:

(11) F44CO2=F12CO2×F16CO2×F16CO2

(12) F45CO2=F13CO2×F16CO2×F16CO2+2×F12CO2×F16CO2×F17CO2

(13) F46CO2=2×F12CO2×F16CO2×F18CO2+2×F13CO2×F16CO2×F17CO2+F12CO2×F17CO2×F17CO2

**Step 8**. Calculate rR45CO2calculated and rR46CO2calculated, ratios calculated from the given δ value for reference peak, for the reference peak. In other words, one must calculate, from the calculations performed so far, what would be the theoretical ratios obtained in Equations (1) and (2), different from what was observed. It is important to note that these calculated ratios now influence the detector amplification, Equations (14) and (15):

(14) rR45CO2calculated=F45CO2F44CO2×100

(15) rR46CO2calculated=F46CO2F44CO2×10003

At this point, one has calculated ratios for masses 45 and 46, based on the abundances and what would be the ideal for the mass balance of the system. However, initially, supporting to Step 1, the observed ratios were previously calculated by the equipment.

**Step 9**. Calculate the correction factors $45_{f}$ and $46_{f},$ respectively, for the masses 45 and 46. This observed difference exists, as the experimental system is not ideal, and it is essential that this non-ideality is included in the calculations, Equations (16) and (17):

(16) 45f=rR45CO2calculatedrR45CO2observed

(17) 46f=rR46CO2calculatedrR46CO2observed

**From the next step, the calculations are performed specifically for each peak**. To provide the uncertainty for each one, we will proceed here using the methane peak just for reference, as the method is the same for any chosen peak.

**Step 10**. Calculate the corrected ratios, R45CO2corrected and R46CO2corrected. The compensation between the calculated and observed ratios will be included in the calculations using a correction factor, calculated as the ratio between the theoretical (numerator) and the observed (denominator), Equations (18) and (19):

(18) R45CO2corrected=rR45CO2calculated×45f100

(19) R46CO2corrected=R46CO2calculated×3×46f1000

**Step 11**. Using the Newton–Raphson method [37], calculate the ratio R18CO2. Before starting the calculation, one removes the influence of the detectors on the obtained reasons. From the mass balances for the ratios R, one can obtain Equation (20):

(20) f=−3K2×(R18CO2)2a+2K×R45CO2corrected×(R18CO2)a+2×R18CO2−R46CO2corrected=0

Note that the only unknown in Equation (20) is R18CO2. Therefore, considering that the equation tends to zero in the solution, and considering that there is only one mathematical solution, one uses the Newton–Raphson method for the calculation of R18CO2, by means of numerical analysis. For that, one has Equation (20) as the objective function (which one wants zero value). One also needs the derivative of this equation in function of the desired variable, in this case, $18_{R}$, given by Equation (21) [34]:

(21) ∂f∂R18CO2=−3K2×2a×(R18CO2)2a−1+2K×R45CO2×a×(R18CO2)a−1+2

Thus, from an initial estimate for (R18CO2)a, *K* being the iteration number in Equation (22), the method is applied until value convergence is reached under a very low desired tolerance, e.g., 10^−1^, to be sure that this numerical step will not represent a significant uncertainty source for the calculation, using Equation (22):

(22) R18CO2K+1=R18CO2K−fK(∂f/∂R18CO2)K

where R18CO2K is the value of R18CO2 in the current iteration, R18CO2K+1 is the value of R to be used in the next iteration, $f^{K}$ is the value of the objective function in the current iteration, and the same goes for the derivative.

**Step 12**. Using the values of the constants *a*, *K* and R18CO2, calculate the ratio R17CO2 accordingly with Equation (23):

(23) R17CO2=K x (R18CO2)a

**Step 13**. Calculate $13_{R}$, Equation (24):

(24) R13CO2=R45CO2corrected−2 x R17CO2

**Step 14.** Finally, calculate the isotopic ratio 13C/12C, Equation (25):

(25) δ13C‰=R13CO2R13CO2/VPDB−1×1000

### 2.4. Uncertainty Evaluation

The combined standard uncertainty is calculated from the expansion of the Taylor series (law of propagation of uncertainties–LPU). Assuming that an output quantity $\hat{y}=f\left(b_{0},b_{1},\ldots ,b_{n}\right)\;$depends on input quantities $b_{0},b_{1},\ldots ,b_{n}$, where each *b_i_* is described by an appropriate probability distribution, the combined standard uncertainty takes the form of Equation (26), taking into account that the quantities are correlated with each other [6]:

(26) $$u_{\hat{y}}^{2}={\sum_{i=1}^{n}\left[\frac{\partial f}{\partial b_{i}}\right]}^{2}u_{i}^{2}+2\sum_{i=1}^{n-1}\sum_{j=i+1}^{n}\frac{\partial f}{\partial b_{i}}\frac{\partial f}{\partial b_{j}}u_{i}u_{j}r_{ij}$$

The type A uncertainty contributions relevant in this study are the standard deviations of the isotopic ratios and the variability of the quantities that are not explicit in the mathematical models, such as gas sample collection pressure, cylinder temperature, reactor temperature, operator, and time. This variability is calculated as a pooled standard deviation, $s_{pooled}$, Equation (27) and expressed in terms of intermediate precision, whose input data come from control charts, available in the Supplementary Materials.

(27) $$s_{pooled}=\sqrt{\frac{\left(n_{1}-1\right)s_{1}^{2}+\left(n_{2}-1\right)s_{2}^{2}+\cdots +\left(n_{m}-1\right)s_{m}^{2}}{n_{1}+n_{2}+\cdots +n_{k}-m}}$$

where *n_i_* is the size of the group and *m* is the number of samples. The type B uncertainty contributions relevant to this study are those related to the constants. From the effective degrees of freedom (number of terms in a sum minus the number of restrictions to the terms of the sum), the appropriate coverage factor, *k*, is calculated in the *t*-Student table, Equation (28) [6]:

(28) $$\upsilon_{eff}=\frac{u_{c}^{4}(\hat{y})}{\sum_{i=1}^{n}\frac{u_{i}^{4}(\hat{y})}{\upsilon_{i}}}$$

Finally, the expanded uncertainty, $U\left(\hat{y}\right),$ is given by Equation (29):

(29) $$U\left(\hat{y}\right)=u_{c}\left(\hat{y}\right)\times k\;(\mathrm{for}\;a\;\mathrm{certain}\;\mathrm{level}\;\mathrm{of}\;\mathrm{confidence})$$

The discussed uncertainty assessment described in this section refers solely and exclusively to analytical uncertainty. The contribution of sampling uncertainty is not part of the scope of this study.

### 2.5. The Use of Measurement Uncertainty in the Assessment of False Compliance Risk

In recent years, the use of uncertainty information in conformity assessment has been widespread in several areas of engineering and science, such as environment pollution [38,39], fuels [40,41,42], biofuels [43], industrial practices [44], drug and medicine analyses [45], pharmaceutical products [46,47], microbiology [48], and radiopharmaceutical activity [49,50].

**Figure 1:**
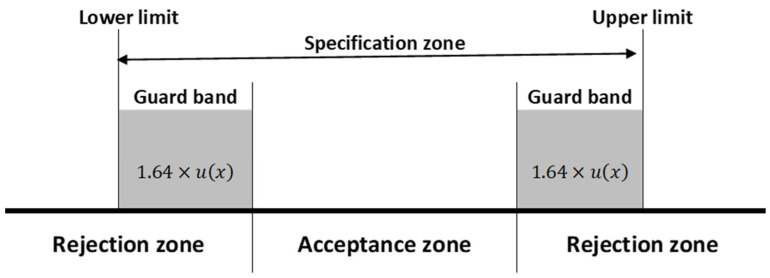
Acceptance and rejection zones for simultaneous upper and lower limits.

A robust and updated approach to solve this issue is the use of guard bands (*g*) [33] that define rejection zones from delineated as specification limit *L* plus a value *g* (1.64 × standard uncertainty for a significance level of 5%). This statistical methodology can be very useful when there is/are partial overlap(s) between the measurement uncertainty and the lower and upper acceptance limits, Figure 1.

This statistical routine can be easily implemented using the Monte Carlo method (MCM), which is based on the generation of random data with known probability distributions.

## 3. Experimental 3.20 × 10^−6^

The initial input data for the calculation of uncertainty considered the isotopic analyses of the secondary standard (Supplementary Materials) and were based on:

(i) Area values and experimental historical data of standard deviations (combined standard uncertainties) of isotopic ratios, uCR45CO2 and uCR46CO2, Table 2:
(ii) Study of variability in terms of intermediate precision whose data come from control charts in the period of July and August 2019. The standard deviations grouped by three different gas cylinders are shown in Table 3:
(iii) Constants, Table 4:

## 4. Results and Discussion

Here, after detailing the calculation of uncertainties, this study compared these values with those in the literature. Finally, the risk of false conformity assessment applied to the isotopic analysis of C1 carbon in the exploratory assessment of natural gas was presented.

### 4.1. Calculation of Expanded Uncertainties by LPU (Law of Propagation of Uncertainties)

To verify the expanded uncertainty, a second internal secondary standard was analyzed, stored in a B1 cylinder of a mixture of 70% mol/mol of methane with 30% mol/mol of ethane, supplied by the company Air Liquide.

The first step is the calculation of the standard uncertainty of the ratios of the CO_2_ experimental areas:

uCR45CO2R45CO22=uA45CO2A45CO22+uA44CO2A44CO22

uCR45CO2=4.69821×10−6

uCrR45CO2=100×u45R=100×4.69821×10−6=0.000469821

uCR46CO2R46CO22=uA46CO2A46CO22+uA44CO2A44CO22

uCR46CO2=3.26629×10−6

uCrR46CO2=10003×uR46CO2=10003×3.26629×10−6=0.001088765

The next step is to calculate the combined standard uncertainty of R13CO2, ucR13CO2 from the available information of $\delta^{13}C$ (certificate) and the ${VPDB}_{R}$ value from the literature. $\delta^{13}C$ value for this standard was −34.4 ± 0.00002, k = 2, for a confidence level of 95.4%.

uc2R13CO2=∂R13CO2∂δ13C×uδ13C2+∂R13CO2∂R13CO2/VPDB×uR13CO2/VPDB2

uc2R13CO2=R13CO2/VPDB1000×uδ13C2+δ13C1000×uR13CO2/VPDB2

ucR13CO2=0.01113761000×0.00012+−34.41000×0.00000162=1.54496×10−6

Calculation of standard uncertainties for carbon abundances, $u_{c}\left(12_{C}\right)\;$and $u_{c}\left(13_{C}\right)$:

ucA13CO2=uR13CO2R13CO2+12=1.54496E−060.010795601+12=1.51213E×10−6=ucA12CO2

Calculation of standard uncertainties for oxygen ratios: ucR17CO2 and ucR18CO2:

uc2R17CO2=∂R17CO2∂R45CO2×uR45CO22+∂R17CO2∂R13CO2×uR13CO22

uc2R17CO2=uR45CO222+−uR13CO222

ucR17CO2=4.69821×10−622+−1.51213×10−622=2.47286×10−6

uc2R18CO2=∂R18CO2∂R17CO2×uR17CO22+∂R18CO2∂K×uK2+∂R18CO2∂a×ua2

uc2R18CO2=R17CO2K1aa×17R×uR17CO22+−R17CO2K1aa×K×uK2+−R17CO2K1a×lnR17CO2Ka2×ua2

ucR18CO2=3.45011×10−40.01027273710.52790.5279×3.45011×10−4×2.47286×10−62+−3.45011×10−40.01027273710.52790.5279×0.010272737×0.000041032+−3.45011×10−40.01027273710.5279×ln3.45011×10−40.0102727370.52792×0.00012=2.6899×10−5

Calculation of standard uncertainties for carbon abundances, ucF16CO2, ucF17CO2 and ucF18CO2:

ucF18CO2=uR18CO218R+12=2.6899×10−50.001488694+12=2.68023×10−5

ucF17CO2=uR17CO2R17CO2+12=2.47286×10−63.45011×10−4+12=2.47105×10−6

uc2F16CO2=uc2F18CO2+uc2F17CO2

ucF16CO2=2.68023×10−52+2.47105×10−62=2.6916×10−5

Calculation of standard uncertainties for carbon abundances, ucF44CO2, ucF45CO2 and ucF46CO2:

uc2F44CO2=∂F44CO2∂F12CO2×u12F2+∂F44CO2∂F16CO2×uF16CO22

uc2F44CO2=(F44CO2)2×uF12CO22+F12CO2×2×F16CO2×uF16CO22

ucF44CO2=0.9980430492×1.51213×10−62+0.98931969915×2×0.998043049×2.6916×10−52=5.31653×10−5

uc2F45CO2=∂F45CO2∂F13CO2×uF13CO22+∂F45CO2∂F16CO2×u16F2+∂F45CO2∂F12CO2×uF12CO22+∂F45CO2∂F17CO2×uF17CO22

uc2F45CO2=(F16CO2)2×u13F2+13F×2×16F+2×12F×17F×u16F2+2×16F×17F×u12F2+2×12F×16F×u17F2

uc2F45CO2=0.9980430492×1.51213×10−62+(0.01068030085×2×0.998043049+2×0.98931969915×0.000344892×2.6916×10−5)2+2×0.998043049×0.000344892×1.51213×10−62+2×0.98931969915×0.998043049×2.47105×10−62

ucF45CO2=5.14008×10−6

uc2F46CO2=∂F46CO2∂F12CO2×uF12CO22+∂F46CO2∂F16CO2×uF16CO22+∂F46CO2∂F18CO2×uF18CO22+∂F46CO2∂F13CO2×uF13CO22+∂F46CO2∂F17CO2×uF17CO22

uc2F46CO2=2×F16CO2×F18CO2×(F44CO2)2×uF12CO22+2×F12CO2×F18CO2+2×F13CO2×F17CO2×uF16CO22+2×F12CO2×F16CO2×uF18CO22+2×F16CO2×F17CO2×uF13CO22+2×F13CO2×F16CO2+F12CO2×2×F17CO2×uF17CO22

uc2F46CO2=2×0.998043049×1.61206×10−3+0.0003448922×1.51213×10−62+2×0.98931969915×1.61206×10−3+2×0.010680301×0.000344892×9.27125×10−52+2×0.98931969915×0.998168627×2.1887×10−52+2×0.998043049×0.000344892×1.51213×10−62+2×0.000344892×0.998043049+0.98931969915×2×0.000344892×2.47105×10−62ucF46CO2=5.29197×10−5

Calculation of standard uncertainties for theoretical CO_2_ areas, ucrR44CO2 and ucrR46CO2:

uCrR45CO2rR45CO22=uF45CO2F45CO22+uF44CO2F44CO22

uCrR45CO21.14867368822=5.14008×10−60.0113196212+5.29197×10−50.9854513882

uCrR45CO2=0.0005254622

uCrR46CO2rR46CO22=uF46CO2F46CO22+uF44CO2F44CO22

uCrR46CO21.0793401762=5.29197×10−50.0031909122+5.31653×10−50.9854513882

uCrR46CO2=0.017907133

Calculation of standard uncertainties for CO_2_ correction factors, $u_{c}\left(45_{f}\right)$ and $u_{c}\left(46_{f}\right)$:

uC45f45f2=urR46CO2calculatedrR46CO2calculated2+urR46CO2observedrR46CO2observed2

$${\left(\frac{u_{C}\left(45_{f}\right)}{1.000097037}\right)}^{2}={\left(\frac{0.0005254622}{1.1486736882}\right)}^{2}+{\left(\frac{0.000469821}{1.148562235}\right)}^{2}$$

$$u_{C}\left(45_{f}\right)=0.000613729$$

uC46f46f2=urR46CO2calculatedrR46CO2calculated2+urR46CO2observedrR46CO2observed2

$${\left(\frac{u_{C}\left(46_{f}\right)}{0.779815704}\right)}^{2}={\left(\frac{0.017907133}{1.079340176}\right)}^{2}+{\left(\frac{0.001088765}{1.384096487}\right)}^{2}$$

$$u_{C}\left(46_{f}\right)=0.012955871$$

Calculation of standard uncertainties for corrected CO_2_ areas, uCR45CO2corrected and uCR46CO2corrected, specifically for methane:

uCR45CO2correctedR45CO2corrected2=urR45CO2calculatedrR45CO2calculated2+u45f45f2

uCR45CO2corrected0.0114139652=0.0005354131.1412857122+0.0006137291.0000970372

uCR45CO2corrected=8.81674×10−6

uCR46CO2correctedR46CO2corrected2=urR46CO2calculatedrR46CO2calculated2+u46f46f2

uCR46CO2corrected0.0030834352=0.0010133511.3180185322+0.0129558710.7798157042

uCR46CO2corrected=5.12965×10−5

Calculation of standard uncertainty for corrected oxygen ratios, $u_{c}\left(18_{R}\right)$:

ucR18CO2=0.000001%×R18CO2=0.00000001×0.001538049=1.53805×10−11

Calculation of the standard uncertainty of $17_{R}$:

uc2R17CO2=∂R17CO2∂K×uK2+∂R17CO2∂R18CO2×uR17CO22+∂R17CO2∂a×ua2

uc2R17CO2=(R18CO2)a×uK2+a x K x (R18CO2)a−1×uR18CO22+K×(R18CO2)a×lnR18CO2×ua2

uc2R17CO2=0.0015380490.5279×0.000041032+0.5279×0.010272737×0.0015380490.5279−1×1.53805×10−112+0.010272737×0.0015380490.5279×ln0.001538049×0.00012ucR17CO2=1.44114×10−6

Calculation of the standard uncertainty of R13CO2:

uc2R13CO2=uR45CO2corrected2+−2×uR17CO22

uc2R13CO2=8.81674×10−62+−2×1.36063×10−72

ucR13CO2=9.27591×10−6 

Calculation of the standard uncertainty $\delta^{13}C\left(‰\right)$:

uc2δ13C‰=∂δ13C‰∂R13CO2×uR13CO22+∂δ13C‰∂R13CO2/VPDB×uR13CO2/VPDB2

uc2δ13C‰=1000R13CO2/VPDB×uR13CO22+−1000×13R(R13CO2VPDB)2×uR13CO2/VPDB2

$$u_{c}^{2}\left(\delta^{13}C\left(‰\right)\right)={\left(\frac{1000}{0.0111376}\times 9.27591\times 10^{-6}\right)}^{2}+{\left(-\frac{1000\times 0.010741425}{0.0111376^{2}}\times 1.60000\times 10^{-6}\right)}^{2}$$

$$u_{c}\left(\delta^{13}C\left(‰\right)\right)=0.844$$

From the information in Table 2, the same calculation is performed for ethane, propane, and CO_2_, Table 5:

The next step is to combine the standard uncertainties of the mathematical models, Table 5, with their respective intermediate precision, Table 3. As predicted in the literature, this latter contribution, the measurement of dispersion, expressed as intermediate precision, is one of the most relevant contributions to the measurement uncertainty [51]. These standard uncertainty values are the final combined standard uncertainties, Table 6.

Finally, the expanded uncertainties are calculated based on Equation (29) for a 95.45% confidence level when considering infinite degrees of freedom (*k* = 2), Equation (27). These uncertainties are shown in Table 7, associated with the δ^13^C values of the compounds of interest.

$$U\left(\delta^{13}C\left(‰\right)\right)=u_{c}\left(\delta^{13}C\left(‰\right)\right)\times k$$

The uncertainty results shown in Table 7 are more significant than that of 0.5‰, historically recommended by the manufacturer of the mass spectrometer for the analytical technique considered in this study. This relative increase suggests greater caution in geochemical interpretations that consider carbon isotopic ratio values using the compounds previously listed.

### 4.2. Comparison with Literature Data

It is important to note that the results of the calculated uncertainties presented in this report, Table 7, comprise two main contributions: one from the equipment and the established procedures, and the other from the calculation procedures outlined in this study.

Regarding natural gas analyses, it is common to spread the information that the measurement uncertainty for carbon isotopes in a natural gas matrix is around 0.5‰. This information is not exactly incorrect, but it is based on an incomplete approach. In fact, the experience of several laboratories shows that this is a reasonable value for a natural gas sample matrix when it is carried out in modern equipment, i.e., gas chromatography–isotope ratio mass spectrometer (GC-IRMS) for the analysis of carbon isotopes performed under minimal quality control conditions. The lack of reliability of this information derives from the fact that this uncertainty ignores a set of error sources that are propagated only after instrumental analysis, which are numerical corrections applied to the measured result.

In many case studies presented in the literature, gases interpreted to have the same origin and/or the same thermal maturity have, at the same time, a difference in their carbon isotopic composition for C_1_ greater than 0.5‰. In the analysis and interpretation of large isotopic gas data sets, interlaboratory variability and other uncertainties (for example, related to gas sampling and handling) are considered to have the least influence on the determination of general isotopic composition trends associated with the various natural geochemical processes [52]. The uncertainty in measuring the carbon isotopic ratios presented here suggests that the geochemical approach generally recognizes a compositional variability that is beyond the commonly reported 0.5‰ value.

In a general way for the considered technique, the possible variabilities that contribute to the analytical uncertainties can be summarized in the following topics [18]: (i) sample: heterogeneity, matrix variations; (ii) sample preparation: weighing (except for gases), extraction, derivatization, collection, storage; (iii) instrumental analysis: conversion to CO_2_, chromatographic separation, transfer through capillaries and valves; (iv) data collection by the equipment: ion current, electronic fluctuations, ion current ratios; (v) integration: software problems, background, time change, baseline choice; (vi) calculation of δ values: correction for ^17^O; (vii) correction on δ values: blank correction, linearity, electronic drift, memory effect; and (viii) scale calibration: adjustment in control chart, normalization procedures.

Comparing the expanded uncertainty calculated in this study (1.7–1.8‰) with the others shown in Table 1, there are clearly higher and lower values. This divergence should be seen with caution since it is not part of a systematic study and has no basis to clarify the differences found, as they may have different sources, such as intrinsic differences between matrices and the calculation methods used by each author, which is fundamental to understand for isotopes and may even differ for instruments of different manufacturers. However, it is worth noting that, of all the studies that applied the expanded methodology and that explicitly reported the inclusion of the uncertainty of the correction methods, only two had the greatest uncertainties found below 1.0‰. It is also important to note that there are other corrections that were not applied in this study, such as the blank corrections or the electronic drift. Their contributions to the accuracy and precision of the work should be appreciated in the future.

The correction method related to the contribution of ^17^O, highlighted in this study, has already been reviewed by several authors [53,54]. There are basically two types of correction: those based on Craig’s method (1957) [55], further developed by Santrock (1985) [31], called SSH, and the method proposed by IUPAC [34]. A review of these methods, including the problems found in the correction approach, is given by [34,53]. The authors detail the limitations of the SSH model and why a linear approximation method would be superior. However, commercial software does not adopt this IUPAC directive—the most recent methodology—and continues to apply the calculation method based on Craig (1957) [55] and Santrock et al. (1985) [35], the oldest method, which apparently introduces greater errors. The discussion of all those methods is beyond the scope of this work. However, this fact must be considered in future studies.

It should also be pointed out that the continuous flow (CF) and dual inlet techniques, both widely used in the literature and in several laboratories currently, have important differences between them. The former leads to higher background values, in addition to other problems, such as the requirement for a reference gas with higher pressure and more residual water in the system, degrading the reproducibility of the results. However, it has advantages that compensate for such limitations, such as the greater ease in the preparation step and injection of samples, the ability to measure more compounds from a complex mixture in the same injection, and the relatively lower required amounts of sample [10,56,57].

The discussions raised so far can be summarized in the following topics: (i) there is no uniformity in the presentation of stable isotope results; (ii) the matrices differences should be discussed considering a physical–chemical approach in such a way that uncertainties, in general, could be better understood and thus lowered; (iii) despite repeated attempts by international bodies such as IUPAC, there is still no consensus about the best ^17^O correction method; (iv) isotopic corrections are not applied in a standardized way. Issues like linearity correction, blank correction, and normalization are applied without clear criteria. Steps that could be followed regardless of the considered isotope technique or analytical laboratory; and (v) the uncertainty arising from calculations and subsequent corrections is often neglected, which does not directly affect the quality control of the analysis laboratory (when not included in the routine), but affects the derived application, such as food quality control or geological interpretation.

From the evaluation and comparison of the results obtained in this study with the ones reported in the literature, one can conclude that the uncertainty values and methodology presented here are reasonable and should be considered when used for interpretations of any nature.

The uncertainty range in the measurement of the carbon isotopic composition in gases (e.g., 1.8‰ for C_1_), although considerably more significant than the one usually reported in the geochemical literature (0.5‰), might have a limited impact on the set of interpretations, generally supported by general trends in the variation of isotopic compositions from many data and case studies.

The typically recognized compositional fields characterized by natural gases from different origins (biogenic, thermogenic, and abiotic) are usually distinguished by their typical isotopic range of compositions. However, it is known that there is an overlapping of isotopic values, and some limits are poorly defined from the typical biogenic (e.g., 70‰), thermogenic (e.g., 40‰) or abiotic (e.g., 10‰) gas compositions (e.g., C_1_ in Etiope and Lollar, 2013) [58]. In these scenarios, either the diagnosis of the origin or the estimates of mixtures between different end members would be affected by such an uncertainty range. In the case of studies involving fractionation factors (e.g., equilibrium temperature), those biases would be more noticeable, making the application tool useless. The uncertainty value of 1.8‰ (for C_1_) should be considered in the geochemical analysis and interpretation, notably in the study of specific cases, such as in areas of exploratory boundaries, where the compositional background of gas occurrences are still unknown or when isotopic measurements plot in intermediate domains, e.g., between −55‰ and −50‰, the approximate limit between biogenic and thermogenic gases [59,60]. In these scenarios, the diagnosis of origin and calculation of the approximate proportions of each contribution can be affected by such an uncertainty range.

### 4.3. Risk of False Compliance Assessment Applied to C_1_ Carbon Isotopic Analysis in Natural Gas Exploratory Evaluation

Here, the information on the measurement uncertainty is used to assess the compliance/non-compliance with the specification. In this study, both methane expanded uncertainties, the current value of 0.5‰ and the proposed value of 1.8‰ (Table 7), are divided by their respective coverage factor and multiplied by 1.64, considering a significance level of 5%.

**Figure 2:**
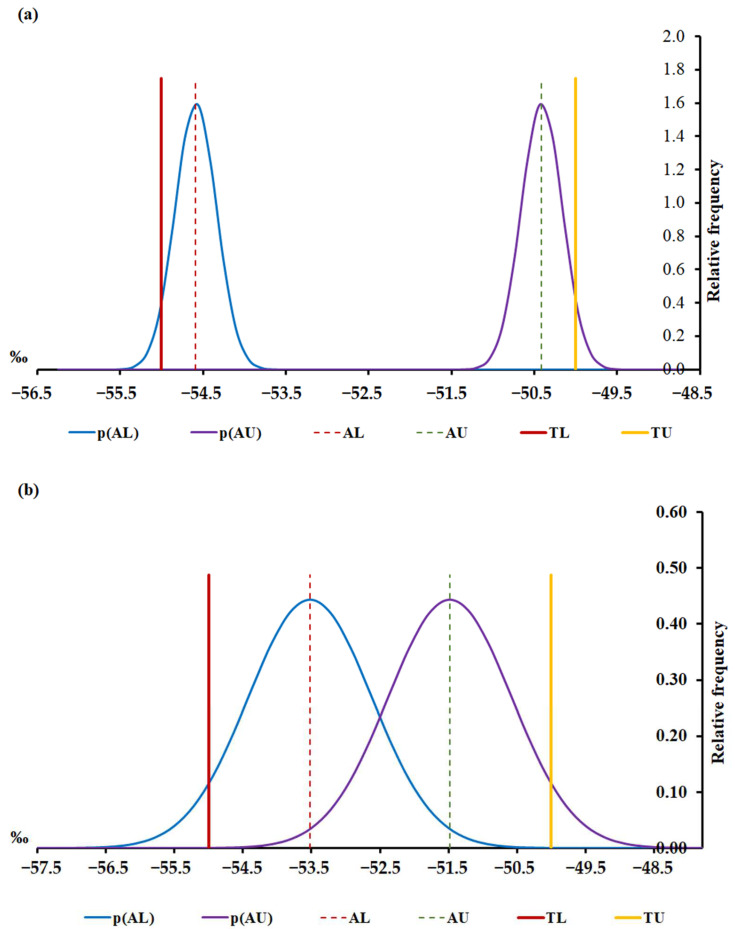
Carbon isotopic analysis of methane conformity assessment with an expanded uncertainty of (**a**) 0.5‰ and (**b**) 1.8‰. p(AL)—probability density at the lower acceptance limit; p(AU)—probability density at the upper acceptance limit; AL—lower acceptance limit; AU—upper acceptance limit; TL—lower tolerance limit and TU—upper tolerance limit.

Histograms with the mean value, its respective uncertainty, guard bands, and lower and upper acceptance limits were calculated. This study used Monte Carlo simulations with 100,000 pseudorandom values for carbon isotopic analysis of methane, and the risk of a false acceptance was assessed (Figure 2).

To guarantee that there were no risks of false compliance assessment applied to carbon isotopic analyses in natural gas exploratory limits, the lower and upper acceptance limits were calculated based on the band guard approach. Considering the underestimated current uncertainty of 0.5‰, the lower and upper acceptance limits were, respectively, −54.6‰ and −50.4‰, Figure 2a; on the other hand, using the proposed realistic uncertainty of 1.8‰, the lower and upper acceptance limits must be more restrictive, i.e., −53.5‰ and −51.5‰, Figure 2b respectively.

## 5. Conclusions

It was possible to increase metrological reliability by means of the determination of the measurement uncertainty associated with the isotopic values reported in this study. It is worth mentioning that the aspects related to (i) gas sampling (in the field), (ii) storage in the lab, (iii) manipulation of the samples by different technicians, and (iv) software operation and data processing are not being considered. This gap suggests a future study in which at least part of these aspects should be considered.

Finally, this study showed that many relatively small measurement uncertainty values are available in the literature without a clear explanation of whether it is exclusively related to the instrumental factors [61] or if it also reflects the uncertainty regarding measurement algorithms.

Furthermore, the measurement uncertainty of the carbon isotopic composition in gases reported here (e.g., 1.8‰ for C_1_), considerably greater than the one generally considered in the geochemical analyses (0.5‰), has an impact on the risk of false compliance assessment of the set of interpretations, generally supported by the general trends of isotopic composition variation using many data and literature case studies.

For these reasons, in the assessment of specific cases, mainly in exploratory frontiers, considering isotopic measurements with values comprised in intermediate compositional domains, and for other applications, e.g., the estimate of temperature equilibration, it is recommended that the uncertainty intrinsic to the calculation method should be incorporated into the corresponding geochemical interpretations.

As for future studies, the contribution of the sampling variability to the measurement uncertainty should be considered since this contribution can significantly affect the risk assessment.

## Figures and Tables

**Table 1:** Published studies associated with isotopic analyses with reported uncertainties (‰) in different matrices.

Reference	Uncertainty Type	Analytical Technique	Calculation Method	Matrix	Uncertainty (‰)
Wong et al., 1995 [7]	Standard deviation	GC-IRMS	Not mentioned	Fatty acids	0.07–0.58
Nørgaard et al., 2002 [8]	Expanded (k = 2)	GC-IRMS	Not mentioned	CO_2_	0.48
Russe et al., 2004 [9]	Expanded complete (k = 2)	GC-IRMS	Several corrections	Standards	0.08–0.25
Boyd et al., 2006 [10]	Standard deviation	GC-IRMS	Not mentioned	C_10_–C_17_	0.10–0.14
Zobitz et al., 2006 [11]	Standard deviation	GC-IRMS	Not mentioned	Air	0.01–0.15
Lollar et al., 2007 [12]	Expanded analytical (k = 2)	FC-IRMS *	Not mentioned	Natural gas	0.4–0.5
Santamaria-Fernandez et al., 2008 [13]	Expanded complete (k = 2)	MC-ICPMS ** and IRMS	SSH	Drug	1.6
Cawley et al., 2009 [14]	Expanded analytical (k = 2)	GC-IRMS	Not mentioned	Steroids	0.5
Munton et al., 2011 [15]	Expanded complete (k = 2)	GC-IRMS	Own 17O correction	Steroids	0.21–1.4
Jones et al., 2013 [16]	Standard deviation	GC-IRMS	Without corrections	Sugars	0.01–0.57
Kornilova et al., 2015 [17]	Standard deviation	GC-IRMS	Several corrections	Volatile organic compounds	0.5
Bulska et al., 2015 [4]	Expanded analytical (k = 2)	GC-IRMS	Not mentioned	Steroids	0.13–0.99
Dunn and Carter, 2018 [18]	Expanded complete (k = 2)	GC-IRMS	Several corrections	Honey	0.084–0.90
Srivastava et al., 2018 [19]	Complete (k = 1)	GC-IRMS	Several corrections	Standards	0.22–0.36
Felix et al., 2019 [20]	95% confidence level	HS-SPME-GC-C-IRMS ***	Two-point correction	Ethanol fuel	2.4–2.5
Strąpoć et al., 2020 [21]	Standard deviation	GC-IRMS	Not mentioned	Natural gas	0.1–5.8
Xue et al., 2021 [22]	Standard deviation	EA-IRMS ^†^	Not mentioned	Organic matter	0.25–0.35
Thomazo et al., 2021 [23]	Standard deviation	GC-IRMS	Without corrections	Carbonates	0.07–1.33
Rampazzo et al., 2022 [24]	Standard deviation	EA-IRMS	Not mentioned	Aqueous samples	0.3–2
Vernooij et al., 2022 [25]	Standard deviation	CF-IRMS	Several corrections	Plants	0.2
Srivastava, 2022 [26]	Standard deviation	DI-IRMS ^‡^	Cross-contamination correction	Isotopic reference materials	0.011–0.021
Day et al., 2022 [27]	Standard error of the mean	IRMS	Multiple point corrections	Eastern rock lobster	0.2
Leitner et al., 2023 [28]	95% confidence level	GC-IRMS	Not mentioned	Chlorinated ethenes	0.2–0.6
Dunn et al., 2015 [29]	Expanded uncertainty (k = 2)	EA-IRMS	Several corrections	Glycine candidate reference material	0.25
Dunn et al., 2015 [30]	Standard uncertainty (k = 2)	EA-IRMS	Several corrections	Primary reference material	27 × 10^−6^

* Continuous flow–isotope ratio mass spectrometry; ** multicollector inductively coupled plasma mass spectrometry; *** headspace solid-phase microextraction gas chromatograph–combustion–isotope ratio mass spectrometry method; ^†^ elemental analyzer-isotope ratio mass spectrometer; and ^‡^ dual-inlet isotope ratio mass spectrometry.

**Table 2:** Area ratios and their respective standard uncertainties.

	R45CO2	$u_{C}$ (R45CO2)	R46CO2	$u_{C}$ (R46CO2)
Reference	0.011486	$4.70\times 10^{-6}$	0.004152	$3.27\times 10^{-6}$
Methane	0.011413	$5.35\times 10^{-6}$	0.003954	$3.04\times 10^{-6}$
Ethane	0.011524	$4.14\times 10^{-6}$	0.003954	$3.20\times 10^{-6}$
Propane	0.011550	$4.15\times 10^{-6}$	0.003980	$3.22\times 10^{-6}$
CO_2_	0.011810	$4.24\times 10^{-6}$	0.003980	$3.22\times 10^{-6}$

**Table 3:** Intermediate precision data.

Methane	Ethane	Propane	CO_2_
$\delta^{13}C$ (‰)			
0.312	0.358	0.346	0.417

**Table 4:** Constants and their respective standard uncertainties.

*a*	0.5279	$u_{a}$	0.0001 [34]
*K*	0.010272737	$u_{K}$	0.00004103 [35]
${VPDB}_{R}$	0.0111376	$u_{{VPDB}_{R}}$	0.0000016 [36]

**Table 5:** Standard uncertainties of mathematical models.

Methane	Ethane	Propane	CO_2_
$u_{c}\left(\delta^{13}C\left(‰\right)\right)$			
0.844	0.792	0.794	0.810

**Table 6:** Final combined standard uncertainties.

Methane	Ethane	Propane	CO_2_
$u_{c}\left(\delta^{13}C\left(‰\right)\right)$			
$\sqrt{0.844^{2}+0.312^{2}}$	$\sqrt{0.792^{2}+0.358^{2}}$	$\sqrt{0.794^{2}+0.346^{2}}$	$\sqrt{0.810^{2}+0.417^{2}}$
0.900	0.870	0.866	0.911

**Table 7:** Carbon isotopic ratio values of C_1_ to C_3_ and CO_2_ and their respective expanded uncertainties.

Methane	Ethane	Propane	CO_2_
δ^13^C (‰)			
−39.2±1.8	−29.1±1.7	−27.1±1.7	−3.7±1.8

## Data Availability

Available data are presented in the manuscript.

## Supplementary Materials

The following supporting information can be downloaded at: https://www.mdpi.com/article/10.3390/molecules29133065/s1. Table S1. The isotopic analyses of the secondary standard–cylinder A. Table S2. The isotopic analyses of the secondary standard–cylinder B. Table S3. The isotopic analyses of the secondary standard–cylinder C.
