# Supplementary material for: Measurement Uncertainty and Risk of False Compliance Assessment Applied to Carbon Isotopic Analyses in Natural Gas Exploratory Evaluation
Source: Molecules. 2024 Jun 27;29(13):3065. doi: 10.3390/molecules29133065 (PMC11243181; doi:10.3390/molecules29133065)
Supplement: Supplementary file 1 [file molecules-29-03065-s001.zip › molecules-3051902-supplementary.pdf]

Table S1. The isotopic analyses of the secondary standard – Cylinder A.

CYLINDER A								
N	Date (DD/MM/YY)	Operator	Shift	Run	Methane $\delta^{13}C$ (‰)	CO_2 $\delta^{13}C$ (‰)	Ethane $\delta^{13}C$ (‰)	Propane $\delta^{13}C$ (‰)
1	16/07/19	Gabriel	Afternoon	LIE08000730	-34.408	-3.570	-29.096	-27.378
2	16/07/19	Gabriel	Afternoon	LIE08000731	-34.316	-3.366	-28.932	-27.501
3	16/07/19	Gabriel	Afternoon	LIE08000732	-34.589	-3.510	-28.817	-27.311
4	16/07/19	Gabriel	Afternoon	LIE08000733	-34.408	-3.497	-28.919	-27.271
5	17/07/19	Gabriel	Morning	LIE08000735	-34.369	-3.554	-28.958	-27.363
6	17/07/19	Gabriel	Morning	LIE08000736	-34.381	-3.518	-28.855	-27.286
7	17/07/19	Gabriel	Morning	LIE08000737	-34.344	-3.452	-28.984	-27.476
8	17/07/19	Gabriel	Morning	LIE08000738	-34.162	-3.572	-28.914	-27.414
9	17/07/19	Gabriel	Afternoon	LIE08000739	-34.348	-3.495	-28.700	-27.395
10	17/07/19	Gabriel	Afternoon	LIE08000740	-34.317	-3.528	-28.822	-27.497
11	17/07/19	Gabriel	Afternoon	LIE08000741	-34.396	-3.599	-28.835	-27.414
12	17/07/19	Gabriel	Afternoon	LIE08000742	-34.212	-3.335	-28.649	-27.377
13	17/07/19	Gabriel	Afternoon	LIE08000743	-34.261	-3.449	-29.048	-27.664
14	18/07/19	Gabriel	Morning	LIE08000744	-34.368	-3.467	-28.918	-27.710
15	18/07/19	Gabriel	Morning	LIE08000745	-34.394	-3.600	-28.940	-27.602
16	18/07/19	Gabriel	Afternoon	LIE08000746	-34.410	-3.596	-29.352	-27.495
17	18/07/19	Gabriel	Afternoon	LIE08000747	-34.351	-3.404	-29.061	-27.488
18	18/07/19	Gabriel	Afternoon	LIE08000748	-34.349	-3.566	-28.863	-27.557
19	18/07/19	Gabriel	Afternoon	LIE08000749	-34.418	-3.559	-28.937	-27.214
20	18/07/19	Túlio	Afternoon	LIE08000750	-34.338	-3.480	-28.904	-27.669
21	18/07/19	Túlio	Afternoon	LIE08000751	-34.317	-3.659	-28.895	-27.588
22	18/07/19	Túlio	Afternoon	LIE08000752	-34.403	-3.524	-28.945	-27.392
23	18/07/19	Túlio	Afternoon	LIE08000753	-34.433	-3.602	-28.929	-27.545

Table S2. The isotopic analyses of the secondary standard – Cylinder B.

CYLINDER B								
N	Date (DD/MM/YY)	Operator	Shift	Run	Methane $\delta^{13}C$ (‰)	CO_2 $\delta^{13}C$ (‰)	Ethane $\delta^{13}C$ (‰)	Propane $\delta^{13}C$ (‰)
1	19/07/19	Gabriel	Morning	LIE08000754	-34.986	-3.777	-29.132	-27.162
2	19/07/19	Gabriel	Morning	LIE08000755	-33.916	-3.299	-28.627	-27.353
3	19/07/19	Gabriel	Morning	LIE08000756	-34.322	-3.355	-28.929	-27.481
4	19/07/19	Gabriel	Morning	LIE08000757	-34.271	-3.298	-28.765	-27.457
5	19/07/19	Gabriel	Afternoon	LIE08000758	-34.028	-3.128	-28.424	-27.108
6	19/07/19	Gabriel	Afternoon	LIE08000759	-34.006	-3.120	-28.389	-27.125
7	19/07/19	Gabriel	Afternoon	LIE08000760	-34.187	-2.993	-28.284	-26.816
8	19/07/19	Gabriel	Afternoon	LIE08000761	-34.184	-2.862	-28.275	-26.890
9	19/07/19	Gabriel	Afternoon	LIE08000762	-33.775	-2.879	-28.179	-27.075
10	19/07/19	Gabriel	Afternoon	LIE08000763	-34.070	-2.769	-28.453	-26.531
11	22/07/19	Gabriel	Morning	LIE08000764	-34.450	-3.610	-28.853	-27.275
12	22/07/19	Gabriel	Morning	LIE08000765	-34.428	-3.526	-28.852	-27.365
13	22/07/19	Gabriel	Afternoon	LIE08000766	-34.366	-3.574	-28.905	-27.351
14	22/07/19	Gabriel	Afternoon	LIE08000767	-34.562	-3.313	-28.979	-27.332
15	22/07/19	Gabriel	Afternoon	LIE08000768	-34.420	-3.370	-28.868	-27.249
16	22/07/19	Gabriel	Afternoon	LIE08000769	-34.343	-3.243	-28.367	-27.175
17	22/07/19	Gabriel	Afternoon	LIE08000770	-34.469	-3.267	-29.068	-27.066
18	22/07/19	Gabriel	Afternoon	LIE08000771	-34.448	-3.252	-28.603	-26.899
19	22/07/19	Gabriel	Afternoon	LIE08000772	-34.380	-3.177	-28.684	-26.968
20	23/07/19	Gabriel	Afternoon	LIE08000773	-34.082	-2.755	-28.342	-26.663
21	23/07/19	Gabriel	Afternoon	LIE08000774	-33.963	-2.778	-28.003	-26.638
22	23/07/19	Gabriel	Afternoon	LIE08000775	-33.819	-2.335	-28.157	-26.389
23	23/07/19	Gabriel	Afternoon	LIE08000776	-33.893	-2.469	-27.946	-26.624
24	23/07/19	Gabriel	Afternoon	LIE08000777	-33.670	-2.361	-27.740	-26.401
25	23/07/19	Gabriel	Afternoon	LIE08000778	-33.608	-2.231	-27.914	-26.274
26	15/08/19	Gabriel	Afternoon	LIE08000831	-34.356	-3.605	-29.002	-27.483
27	15/08/19	Gabriel	Afternoon	LIE08000832	-34.566	-3.517	-28.555	-27.230
28	15/08/19	Gabriel	Afternoon	LIE08000833	-34.502	-3.615	-29.033	-27.643
29	15/08/19	Gabriel	Afternoon	LIE08000834	-34.459	-3.473	-28.796	-27.493
30	15/08/19	Gabriel	Afternoon	LIE08000835	-34.583	-3.543	-28.898	-27.517
31	16/08/19	Gabriel	Morning	LIE08000836	-34.483	-3.509	-28.861	-27.249
32	16/08/19	Gabriel	Morning	LIE08000837	-34.328	-3.480	-28.899	-27.232
33	16/08/19	Gabriel	Afternoon	LIE08000841	-34.513	-3.380	-28.818	-27.197
34	16/08/19	Gabriel	Afternoon	LIE08000842	-34.538	-3.304	-28.746	-27.312
35	16/08/19	Gabriel	Afternoon	LIE08000843	-34.502	-3.322	-28.709	-27.128
36	16/08/19	Gabriel	Afternoon	LIE08000844	-34.502	-3.384	-28.816	-27.187
37	16/08/19	Gabriel	Afternoon	LIE08000845	-34.345	-3.288	-28.724	-27.299
38	16/08/19	Gabriel	Afternoon	LIE08000846	-34.444	-3.205	-28.860	-27.075

Table S3. The isotopic analyses of the secondary standard – Cylinder C.

CYLINDER C								
N	Date (DD/MM/YY)	Operator	Shift	Run	Methane $\delta^{13}C$ (‰)	CO_2 $\delta^{13}C$ (‰)	Ethane $\delta^{13}C$ (‰)	Propane $\delta^{13}C$ (‰)
1	24/07/19	Gabriel	Afternoon	LIE08000779	-34.606	-3.700	-28.916	-27.547
2	24/07/19	Gabriel	Afternoon	LIE08000780	-34.534	-3.501	-28.869	-27.473
3	24/07/19	Gabriel	Afternoon	LIE08000781	-34.552	-3.315	-28.901	-27.141
4	24/07/19	Gabriel	Afternoon	LIE08000782	-34.464	-3.324	-28.946	-27.184
5	25/07/19	Gabriel	Morning	LIE08000784	-33.809	-2.614	-27.964	-26.880
6	25/07/19	Gabriel	Morning	LIE08000785	-33.802	-2.546	-28.275	-26.685
7	25/07/19	Gabriel	Morning	LIE08000786	-33.734	-2.412	-28.046	-26.639
8	25/07/19	Gabriel	Morning	LIE08000787	-33.875	-2.526	-28.130	-26.738
9	25/07/19	Gabriel	Morning	LIE08000788	-33.879	-2.491	-28.074	-26.464
10	25/07/19	Gabriel	Afternoon	LIE08000789	-33.821	-2.408	-27.991	-26.529
11	25/07/19	Gabriel	Afternoon	LIE08000790	-33.789	-2.268	-27.951	-26.310
12	25/07/19	Gabriel	Afternoon	LIE08000791	-33.600	-2.313	-27.900	-26.467
13	25/07/19	Gabriel	Afternoon	LIE08000792	-33.773	-2.420	-28.092	-26.509
14	25/07/19	Gabriel	Afternoon	LIE08000793	-33.706	-2.330	-27.862	-26.318
15	25/07/19	Gabriel	Afternoon	LIE08000794	-33.801	-2.444	-28.001	-26.150
16	25/07/19	Gabriel	Afternoon	LIE08000795	-33.737	-2.150	-27.642	-26.565
17	25/07/19	Gabriel	Afternoon	LIE08000796	-33.685	-2.129	-27.803	-26.263
18	25/07/19	Gabriel	Afternoon	LIE08000797	-33.540	-2.067	-27.730	-26.382
19	25/07/19	Gabriel	Afternoon	LIE08000798	-33.562	-2.131	-27.811	-26.269
20	30/07/19	Gabriel	Afternoon	LIE08000803	-34.585	-3.468	-29.095	-27.607
21	30/07/19	Gabriel	Afternoon	LIE08000804	-34.160	-3.574	-28.916	-27.413
22	30/07/19	Gabriel	Afternoon	LIE08000805	-34.377	-3.521	-28.906	-27.333
23	31/07/19	Gabriel	Morning	LIE08000806	-34.227	-3.297	-28.582	-26.887
24	31/07/19	Gabriel	Morning	LIE08000807	-34.189	-3.139	-28.822	-27.116
25	31/07/19	Gabriel	Morning	LIE08000808	-34.229	-3.120	-28.555	-27.272
26	31/07/19	Gabriel	Morning	LIE08000809	-34.488	-3.478	-28.937	-27.300
27	31/07/19	Gabriel	Afternoon	LIE08000810	-34.147	-3.603	-28.771	-27.105
28	31/07/19	Gabriel	Afternoon	LIE08000811	-34.574	-3.534	-28.687	-27.449
29	31/07/19	Gabriel	Afternoon	LIE08000812	-34.604	-3.553	-28.946	-27.545
30	31/07/19	Gabriel	Afternoon	LIE08000813	-34.434	-3.326	-28.971	-27.092
31	31/07/19	Gabriel	Afternoon	LIE08000814	-34.571	-3.544	-29.038	-27.503
32	31/07/19	Gabriel	Afternoon	LIE08000815	-34.644	-3.597	-28.660	-27.038
33	02/08/19	Gabriel	Morning	LIE08000816B	-34.061	-2.929	-28.380	-26.605
34	12/08/19	Gabriel	Afternoon	LIE08000820	-34.998	-3.363	-28.836	-26.932
35	12/08/19	Gabriel	Afternoon	LIE08000821	-34.561	-3.375	-28.726	-27.051
36	12/08/19	Gabriel	Afternoon	LIE08000822	-34.635	-3.447	-28.563	-27.163
37	12/08/19	Gabriel	Afternoon	LIE08000823	-34.701	-3.387	-28.696	-27.174
38	12/08/19	Gabriel	Afternoon	LIE08000824	-34.542	-3.459	-28.776	-26.943
39	13/08/19	Gabriel	Morning	LIE08000826	-34.423	-3.075	-28.530	-26.944
40	13/08/19	Gabriel	Afternoon	LIE08000827	-34.480	-3.278	-28.600	-26.854
41	13/08/19	Gabriel	Afternoon	LIE08000828	-34.645	-3.153	-28.593	-26.976
42	15/08/19	Gabriel	Afternoon	LIE08000830	-33.989	-2.473	-27.962	-26.337

43	16/08/19	Gabriel	Afternoon	LIE08000838	-34.418	-3.494	-28.776	-27.138
44	16/08/19	Gabriel	Afternoon	LIE08000839	-34.462	-3.377	-28.620	-27.344
45	16/08/19	Gabriel	Afternoon	LIE08000840	-34.485	-3.299	-28.667	-27.327
